# Electride Formation of HCP‐Iron at High Pressure: Unraveling the Origin of the Superionic State of Iron‐Rich Compounds in Rocky Planets

**DOI:** 10.1002/advs.202308177

**Published:** 2024-04-12

**Authors:** Ina Park, Yu He, Ho‐kwang Mao, Ji Hoon Shim, Duck Young Kim

**Affiliations:** ^1^ Center for High‐pressure Science and Technology Advanced Research (HPSTAR) Shanghai 201203 China; ^2^ Department of Chemistry Pohang University of Science and Technology Pohang 37673 Republic of Korea; ^3^ Key Laboratory of High‐Temperature and High‐Pressure Study of the Earth's Interior Institute of Geochemistry Chinese Academy of Sciences Guiyang 550081 China; ^4^ Department of Physics Pohang University of Science and Technology Pohang 37673 Republic of Korea; ^5^ Division of Advanced Nuclear Engineering Pohang University of Science and Technology Pohang 37673 Republic of Korea

**Keywords:** Earth's inner core, electride, hcp iron, iron hydride, superionicity

## Abstract

Electride possesses electrons localized at interstitial sites without attracting nuclei. It brings outstanding material properties not only originating from its own loosely bounded characteristics but also serving as a quasiatom, which even chemically interacts with other elemental ions. In elemental metals, electride transitions have been reported in alkali metals where valence electrons can easily gain enough kinetic energy to escape nuclei. However, there are few studies on transition metals. Especially iron, the key element of human technology and geophysics, has not been studied in respect of electride formation. In this study, it is demonstrated that electride formation drives the superionic state in iron hydride under high‐pressure conditions of the earth's inner core. The electride stabilizes the iron lattice and provides a pathway for hydrogen diffusion by severing the direct interaction between the metal and the volatile element. The coupling between lattice stability and superionicity is triggered near 100 GPa and enhanced at higher pressures. It is shown that the electride‐driven superionicity can also be generalized for metal electrides and other rocky planetary cores by providing a fundamental interaction between the electride of the parent metal and doped light elements.

## Graphical abstract

A superionic state, a solid lattice with liquid‐like mobile ions usually at high PT conditions, is a possible state of the Earth's interior. The study proposes an electride‐driven scenario for the origin of superionicity in the Earth's inner core, where an electride, localized electron at interstitial sites, offers a pathway toward the decoupling between the mobile light‐element ions and stable iron lattice.

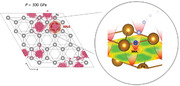

## 1 Introduction

Electrides are electrons occupying the interstitial sites and playing a role as anions. These electrons are localized at so‐called non‐nuclear attractor (NNA) sites, the sites with high electron‐localizability produced by Pauli's exclusion principle and Coulombic repulsion competing with the kinetic energy.^[^ ^1^, ^2^, ^3^, ^4^, ^5^, ^6^, ^7^, ^8^ ^]^ These localized electrons generate novel properties due to their loosely binding characteristics, so potential applications such as catalysts, reducing agents, electron emitters, battery anodes, and even superconductors have been proposed and discovered.^[^ ^9^, ^10^, ^11^, ^12^, ^13^, ^14^, ^15^, ^16^, ^17^, ^18^ ^]^

Electrides were commonly discovered in materials containing alkali metal or alkaline earth metal elements, which can easily offer electrons to become cationic. On the other hand, high‐pressure conditions can also be one of the intrinsic platforms to induce an electride state, because valence electrons gain enough kinetic energy to escape the attracting potential well of the atomic nuclei and occupy vacant space in solids. This mechanism was theoretically modeled by comparing the energy between the valence orbital of metal ions and *s*‐like NNA orbitals,^[^ ^7^ ^]^ and computationally verified for real metals, for example, by predicting open‐structured aluminum.^[^ ^19^ ^]^ Furthermore, a formation of open‐structured magnesium was recently observed at tera‐pascal pressure with the presence of NNA experimentally.^[^ ^20^ ^]^ Interestingly, these works imply that NNA can play a structural role as a real anion.

On the other hand, the chemical and physical role of NNA on structure and material properties was found to be more intricate and prominent when it meets hydrogen atoms. Since the relationship between a hydrogen atom and NNA was proposed by Savin et al.,^[^ ^21^ ^]^ many studies have shown indirect clues about the relationship between the two. It was found that the structural, electronic, and dynamic properties of doped atoms were governed by the NNAs of the parent compound.^[^ ^22^, ^23^, ^24^, ^25^ ^]^ The study,^[^ ^25^ ^]^ particularly regarding the relationship between the electride and superionic state, implies that the NNA may affect not only the electronic property but also the energetics and kinetics of the material. However, to date, few studies have discovered the coexistence of both electride and superionic states,^[^ ^16^, ^25^ ^]^ and the relationship between the two states has been even more rarely discussed.

In this work, we found that the high‐pressure superionic behavior of H anions in *hcp*‐FeH_x_ (x < 1)^[^ ^26^ ^]^ can be a comprehensive example to study both superionicity and electride phenomena and their coupling. Iron‐light‐element alloy, *hcp*‐Fe(H, C, or O)_x_ (x < 1), is one of the Earth's inner core (IC) material candidates, and it becomes superionic under a high‐pressure condition of ≈300 GPa. In a superionic state, hydrogen ions can freely diffuse while the *hcp* iron lattice remains dynamically stable. A superionic state is widely reported to exist in planetary interiors with significant influence on the properties of interior materials such as seismic velocity, electrical conductivity, and magnetism^[^ ^26^, ^27^, ^28^, ^29^ ^]^ due to light ion diffusion.

We found that it also becomes an electride at high‐pressure conditions of ≈100 GPa and that the doped hydrogen ions were governed by the NNAs of the parent compound *hcp*‐Fe. The hydrogen ions interact considerably weakly with iron atoms, which is unusual considering the extremely high‐pressure conditions. By examining the stabilization effect of NNA to maintain the metal lattice, we propose that NNA can play a role in offering a pathway to superionicity.

## 2 Possible Entanglement of Electride and Superionic State

**Figure 1:**
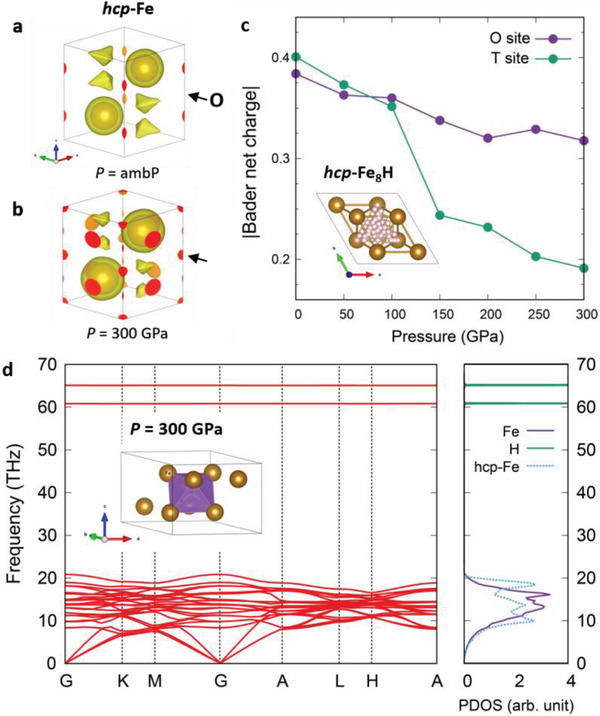
a,b). Electron localization function (ELF) isosurface of pure *hcp*‐Fe at ambient pressure and *P* = 300 GPa, respectively (with isosurface cutoff of 0.35). c). Absolute Bader net charge of H‐ions in *hcp*‐Fe_8_H as a function of pressure. Purple and green circles represent the hydrogen anion doped at octahedral (H^O^) and tetrahedral (H^T^) sites. (The inset visualizes all the positions of calculated H‐ions with Fe─H distance larger than 1 Å.) d). Phonon band structures and partial density of states (solid lines) of Fe_8_H^O^ at 300 GPa. The cyan dashed curve is the total density of states of pure *hcp*‐Fe at 300 GPa as a comparison.

We start by looking at how the NNA develops as pressure increases in *hcp*‐Fe and how it offers a pathway toward the superionic state of *hcp*‐Fe_8_H. For *hcp*‐Fe, the localizability of interstitial electrons changes as pressure increases, as shown in **Figure** **1**a,b. At ambient pressure, there is no electron localization function (ELF) blob at the octahedral (O) sites − (0, 0, 0.5), for example, which is the isosurface of ELF with a certain criterion value of 0.35, but it clearly appears at 300 GPa, as marked by arrows. (ELF contour plot is shown in Figure S1 (Supporting Information), and the list of O and the other representative interstitial sites is given in Table S1, Supporting Information.) This ELF blob implies the formation of NNAs, the key signature of an electride. We want to note that the degree of localization of iron charge density is weaker than the other archetypal high pressure electrides, for example, magnesium in the same *hcp* structure. Iron has more itinerant electron density and hence relatively hard to be cationic than magnesium. Especially for high pressure conditions, a huge difference in the decrease of the electronegativity between magnesium and iron^[^ ^30^ ^]^ partially elucidates why magnesium shows a much stronger electride behavior than iron. But there's a clear trend that if we increase the pressure further, the size of the ELF blob at the O site increases while that at the T site decreases. It indicates that while the ELF value at the O site increases as pressure increases, that at the T site decreases. It is also known that in the case of iron *d*‐electrons, the charge density accumulation is not significant possibly due to the stabilization of *d* orbitals at high‐pressure conditions against the NNA orbitals.^[^ ^7^ ^]^ When hydrogen atoms are doped into *hcp*‐Fe, nevertheless, the responses of the O sites with the NNA orbital and those of the other interstitial sites are significantly different. Since NNA can interact with and provide electrons to a hydrogen atom (22‐25), the formation of NNA can also be chased by observing the Bader net charge of a hydrogen atom as pressure changes.

The Bader net charge of hydrogen ions doped at all possible interstitial positions with the Fe─H distance larger than 1 Å is investigated. (All interstitial positions are described in the inset of Figure 1c.) Here, Fe_8_H, the 2×2×1 supercell of *hcp* iron with one hydrogen atom, is used. The full results are shown in Figure S2 (Supporting Information), and the results of octahedral (O) − now become (1/2, 1/2, 1/2) for Fe_8_H – and tetrahedral (T) – (2/3, 1/3, 5/8) – sites as the most representative cases are shown in Figure 1c. Here, the absolute value of the net charge was plotted, and hydrogen ions have a negative net charge, so they are anions. Near ambient pressure, the Bader net charge of H^O^ and H^T^ – hydrogen ion doped at the O and T sites, respectively – does not differ much, and the H^T^ has a slightly larger charge possibly due to the shorter Fe─H bond length despite the smaller spatial size. However, H^O^ begins to possess a larger charge than H^T^, as the pressure exceeds 100 GPa. The decreasing slope also significantly differs, which means that the Bader net charge of H^O^ is more tolerant of pressure change than that of the H‐ions in the tetrahedral or the other sites, where the electrons escape rapidly under pressure. This remarkable difference between H^O^ and H^T^ is due to the formation of the NNA at the O site at high‐pressure conditions, where the transition point of NNA formation can be represented with the crossing point of Bader net charges at ≈100 GPa. The decreasing trend of the Bader net charges can be understood by the competition between the iron *d*‐orbitals and *s*‐like NNA orbitals. Since the level of *d*‐orbitals is less sensitive to pressure than that of *s*‐ or *p*‐orbitals, electrons stay more likely in the iron *d*‐orbitals as pressure increases.^[^ ^7^ ^]^

In addition, for all H positions tested, the Bader net charge is predominantly correlated to the distance from the O site. As shown in Figure S2 (Supporting Information), the Bader charge decreases rapidly as the H ion moves away from the O site, especially when the H‐ions are at z = 0.5 plane. Such correlation gets stronger as pressure increases. At ambient pressure, some points can have a similar Bader net charge as H^O^ or an even higher charge, on the other hand, at 300 GPa, all points have a smaller charge than H^O^. These indicate that *hcp*‐Fe (and *hcp*‐Fe_8_H) shows the signatures of the electride state with NNAs formed at the O site at high‐pressure conditions. As in previous works on electride,^[^ ^12^, ^18^, ^31^ ^]^ we will use the term for the electride state as Fe^δ +^ (δ*e* ^−^), which also can be abbreviated as Fe: *e* ^−^ for the sake of simplicity. Here, δ indicates the slight charge accumulation at the O site. For the hydrogen‐doped case, we will also use FeH_*x*_: *e* ^−^ (*x* < 1) to notate both the sub‐stoichiometric hydrogen and the remaining NNAs at vacant O sites. We also note that the dominance of the Bader net charge at the O site was observed even for the case with higher H content, for example, Fe_4_H.

The phonon and electronic structures of Fe_8_H^O^: *e* ^−^, or ${\mathrm{FeH}}_{0.125}^{O}:e^{-}$ equivalently, can elucidate the relationship between the electride and the superionicity. With the NNA formed at 300 GPa, the doped H anion displays flat phonon bands for all translational modes over the whole Brillouin zone, as shown in Figure 1d. These highly localized vibrational modes indicate that the H atom is under extremely weak interaction potential with *non‐bonding* characteristics, that is, behaves like a rattler.^[^ ^32^, ^33^ ^]^ As shown in the partial phonon density of states (PDOS) in Figure 1e, these flat modes do not contain Fe contribution, and Fe PDOS also does not have any contribution from H atoms. The independence of the phonon modes between neighboring Fe and H atoms is peculiar, and it implies that the H anion can freely move with the shallow harmonic potential well of the Fe: *e* ^−^ lattice. The stable iron lattice maintained by the presence of NNA while the mobile H‐ions freely come in and out of the NNA sites is nothing but the superionic state. The flat H‐driven phonon bands and the PDOSs of Fe and H independent to each other can then be the prerequisite of the superionic state, and the role of the NNA for structural stability is necessary. Such role of the NNA was partially discussed and observed for the open structure metal at high‐pressure conditions.^[^ ^19^, ^20^ ^]^ We also checked that the NNAs were stable regardless of the second doping in other O sites, and the possible coupling between NNA and H was similarly proposed in other works.^[^ ^24^ ^]^

**Figure 2:**
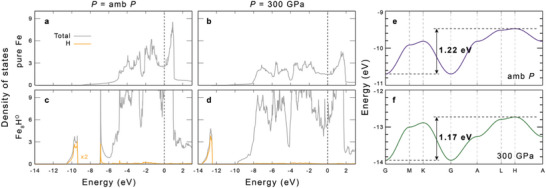
a–d). The density of states of pure *hcp*‐Fe (a‐b) and Fe_8_H^O^ (c‐d) at ambient pressure and 300 GPa. e,f). Band structure of H 1s states at ambient pressure (e) and 300 GPa (f). The bandwidth is indicated with an arrow and its value.

From the evolution of the electronic structures under pressure, we can partially understand the non‐bonding nature of the interaction between Fe and H. We found that the H anion reflects the localized characteristics of the NNA so that becomes electronically separated from the Fe atoms. As shown in **Figure** **2**, the bandwidth of H 1s states does not increase as pressure increases from ambient pressure to 300 GPa. Rather it decreases by ≈50 meV (−4%) while the bandwidth of Fe states increases by ≈4 eV (+70%), which is unusual considering the extreme pressure condition. In addition, at ambient pressure, there is a finite H contribution to the Fe bands ≈ −7 eV with a sharp peak structure due to the flatness around the Γ point. Interestingly, this hybridization behavior completely disappears at 300 GPa. We expect that it is deeply related to the localized characteristic of the NNA and its coupling to H‐ions so that H‐ions become more localized at higher pressure conditions, which also reinforces the non‐interacting behavior between H and Fe ions. This can play an important role in maintaining the stable FeH_*x*_: *e* ^−^ lattice. In this picture of the possible entanglement between the electride and the superionic state, the NNA directly interacts with the H atom and provides a localizable electron. Hence, a direct Fe─H bond or the change of a Fe‐Fe bond is unnecessary for becoming a charged H anion. As a result, the harmonic potential of the *hcp* iron lattice strengthened by the NNA remains independent of the doped H anion and stable under its diffusion.

## 3 Evolution of Superionic State under Pressure

**Figure 3:**
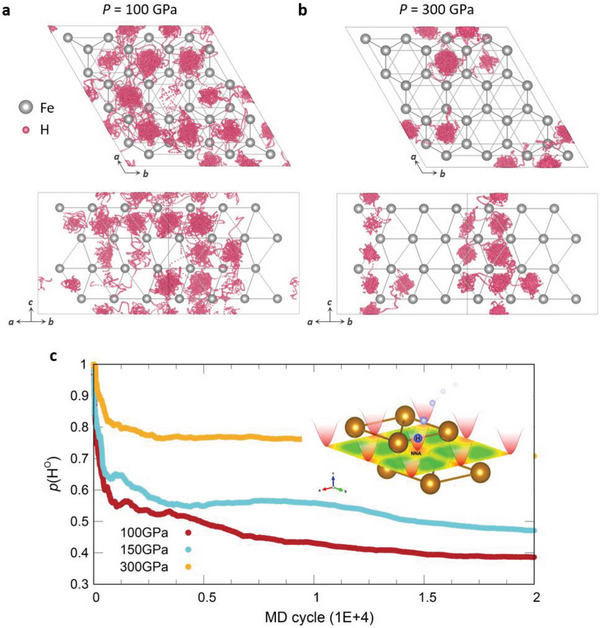
a,b). Molecular dynamics (MD) trajectory of one H anion for superionic *hcp*‐FeH_0.25_ state at *P* = 100 and 300 GPa with the isothermal condition, respectively. (*T* = 3000 K) Here, we draw Fe atoms as fixed reference points since they harmonically vibrate. c). The portion of H anions (*p*(H^O^)) near the octahedral sites as a function of MD cycles at *P* = 100, 150, and 300 GPa. The criteria are set as 0.3 · (Fe─H bond length at each pressure condition) of the distance between the H anion and the position of the O site (0.5, 0.5, 0.5). Inset describes the diffusion process of H anion around the NNA site schematically (2D display of ELF indicates the NNA formation at the octahedral sites marked with dashed hexagons).

Next, the pressure evolution of the superionic state at isothermal conditions was examined using the ab initio molecular dynamics (AIMD) simulation to be compared with the electride behavior. 4×4×2 supercell structure of *hcp*‐FeH_0.25_ was investigated at *T* = 3000 K and *P* = 15, 100, 150, and 300 GPa conditions. As shown in **Figure** **3**a,b, H anions are more captured around the O sites as pressure increases, consistent with the formation of the NNA at the O sites. At 100 GPa, H anions are distributed relatively sparsely throughout the whole cell. On the other hand, at 300 GPa, H anions are observed mainly around the O sites. (There are also shown the anisotropy of diffusion trajectory,^[^ ^34^ ^]^ and we will discuss it in detail later.) To quantify this difference, the portion of H anions near the O sites (i.e., *p*(H^O^) = *n*(H^O^)/*n*(H^tot^)) as a function of the number of cycles is plotted in Figure 3c. Here, the H anion near the O sites was defined as the ion with the distance from O sites smaller than 0.3 times of neighboring Fe─H distance. The *p*(H^O^) rapidly decreases at the beginning as H anions quickly diffuse into the cell, but soon it converge to a constant. As the pressure increases, the converged *p*(H^O^) value increases significantly – 38.61%, 47.11%, and 70.80% for 100, 150, and 300 GPa, respectively. A different distance cutoff was also verified, for example, 0.5 times of Fe─H distance, which shows the same trend.

It indicates how much O sites are thermodynamically favored. In other words, the H anion at the NNA site is energetically more stable at higher pressure conditions. The inset of Figure 3c schematically describes the motion of H anions under harmonic potential well near the O site. The NNAs are formed at O sites of the *hcp* structure at above 100 GPa, and they offer an attractive potential well for the H anions so that they mostly reside there. But the potential well is shallow due to the non‐bonding characteristics so that H anions either stay there or easily diffuse to other O sites. Also, adding and removing the H anion does not affect the stability of the *hcp* cell, as will be discussed in the next part. It is worth noting that the low‐pressure condition of 15 GPa was also examined, but the *hcp* cell completely collapsed. This pressure condition was in a range where the NNA could not be observed.

## 4 Potential Energy Surfaces Evolution under Pressure

**Figure 4:**
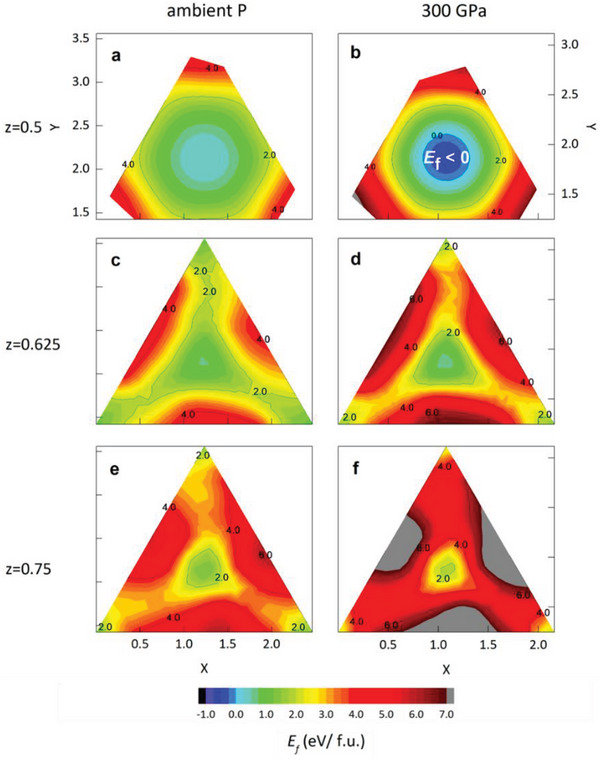
a–f). Potential energy surface for z = 0.5, 0.625, and 0.75 planes of *hcp*‐Fe_8_H for the pressure condition of ambient pressure (left column) and *P* = 300 GPa (right column). The color bar indicates the formation energy in eV/f.u., and the positions of the calculated H anions are depicted in the inset of Figure 1c.

The potential energy surfaces (PES) obtained from the DFT calculation elucidate the above MD results. As shown in **Figure** **4**, the PES for z = 0.5, 0.625, and 0.75 lattice planes were obtained by moving an H atom as a probe in the iron sublattice. The contour plot represents the formation energy referenced by the solid or gaseous hydrogen and iron.^[^ ^35^, ^36^ ^]^ First, we could see that the H ion can be stabilized when it is bound to the NNA. The formation energies at ambient pressure are all positive, explaining the thermodynamic instability of the *hcp* lattice under hydrogen doping. At 300 GPa, the formation energy for all positions increases further as pressure increases, while only the O site shows the opposite trend to become negative, as marked in Figure 4b. It is noteworthy that the formation energy at the O site starts to become negative near the pressure condition of 150 GPa. (See Table S2, Supporting Information for the formation energy at different pressure conditions.) Contrary to the usual expectation that the densely packed iron atoms can hardly bear H anions at high‐pressure conditions, our result shows the stabilization of H anions at the O site. As observed from the Bader net charge results above, the empty NNA orbitals become active under H doping, giving a higher Bader net charge than other interstitial sites. The coupling between the NNA and H atom seems to stabilize the local charge at the O site so that the formation energy becomes negative.

The PES also elucidates the anisotropic diffusion pathway. There is a smaller energy difference between the in‐plane (xy) and out‐of‐plane diffusion pathways at ambient pressure. But at 300 GPa, the barrier from the O sites to the triangular corner becomes higher to restrict the in‐plane diffusion of H anions. It is well captured by looking at the z = 0.625 and z = 0.75 planes. The potential energy curve for three representative diffusion paths is also shown in Figure S4 (Supporting Information). Three paths connect the O site at (0.5, 0.5, 0.5) and the nearest stable O or T sites. The kinetic energy barrier for the path from one O site at z = 0.5 plane (O1) to the next O site at z = 1.0 plane (O2) is the lowest, and the pressure further increases the relative energy barrier difference compared to the other paths. At 300 GPa, for example, the kinetic barrier of the O1‐O2 path is twice as small as that of the second probable O–T path. This is consistent with the anisotropic H diffusion observed in MD simulation at 300 GPa, as the H anion moves around the O site and then predominantly diffuses along the c axis to the next O sites. It rarely diffuses along the *ab* plane, unlike the 100 GPa case.

As indicated from the potential energy surface, the pressure makes the O site doping more stable than other sites. The energy barrier for the diffusion also increases as pressure increases, so the critical temperature of the superionic transition should increase. This implies that even though the formation of the NNA may help establish the superionic state of iron‐light‐element alloy, it does not result in faster diffusion. This expected phenomenon was also observed in the MD simulation under IC conditions, as the diffusion coefficient decreases at higher pressure conditions for the case of FeH_0.25_ and FeO_0.0625_ at the same temperatures.^[^ ^26^ ^]^ We also note that the formation energy at 360 GPa, which corresponds to the exact IC pressure condition, is ≈ −840 meV/f.u., more than twice that at 300 GPa, which is ≈ −340 meV/f.u.

## 5 Effects of Secondary Metal or Different Anionic Elements

**Figure 5:**
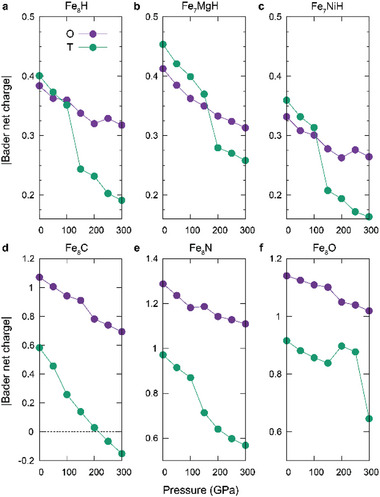
Evolution of the Bader net charge as a function of the pressure of a). *hcp*‐Fe_8_H, with partial substitution of the secondary metal element Mg or Ni – b). Fe_7_MgH and c). Fe_7_NiH, and with anion substitution to carbon, nitrogen, or oxygen – d). Fe_8_C, e). Fe_8_N, and f). Fe_8_O, respectively. Purple and green circles are the Bader net charge of the O and T sites, respectively.

To take a more realistic environment of IC into account, the partial substitution of secondary metal elements such as Ni or Mg was investigated.^[^ ^37^ ^]^ As shown in **Figure** **5**b,c, both Fe_7_MgH and Fe_7_NiH show the same trend of Bader net charge evolution – H^O^ and H^T^ have almost the same Bader net charge and decreasing slope at the low‐pressure regime but a significant difference at the high‐pressure regime. However, the extent of the Bader net charge differs, as Mg substitution gives a larger net charge while Ni gives a slightly smaller net charge. This is because *p*‐electrons of Mg produce much stronger NNA and a higher possibility of charge accumulation at the O sites.^[^ ^8^ ^]^ On the other hand, the transition point toward the superionic state, that is, the crossing point of two curves in the graph, also shifts to a higher pressure value by ≈ 50 GPa in the case of Mg substitution. We speculate that Mg atoms easily give electrons to both O and T sites at relatively lower pressure conditions, so two sites compete. In the case of Ni, that transition point does not seem to change.

The effects of other anions, such as carbon, nitrogen, and oxygen, were also investigated. These volatile elements are also possible constituents in IC, and the stable *hcp* structure above 100 GPa and the possible superionic state were reported.^[^ ^26^, ^38^, ^39^, ^40^ ^]^ As shown in Figure 5d–f, all anions have larger Bader net charges when doped at the O site at ambient pressure, unlike the hydrogen‐doped case. Hence, it is hard to see a noticeable crossover of two net charge curves as dramatic as the H case, but the decreasing slopes are still significantly different. As seen from the ELF evolution as depicted in Figure S5 (Supporting Information), a larger difference in Bader net charge implies the formation of the NNA at the O site with increasing pressure. Lastly, the evolution of the potential energy curve along the O–T diffusion path under pressure changes is shown in Figure S6 (Supporting Information), which is also consistent with the H‐doped case– the O site becomes more stabilized, and the barrier energy becomes anisotropic as pressure increases.

## 6 Discussion and Conclusion

We suggest that the NNA phenomena and the volatile diffusion are intertwined. It would also give the general implication for doped metal electrides including rocky planetary cores since this relationship originated from the intrinsic property of the parent metal, iron. In addition, our study provides an in‐depth understanding of the chemical interaction between the NNA, that is, localized electrons, and doped volatile elements. It not only provides a new clue about the superionic transition under pressure but also implies the role of NNA in the dynamical properties of electrides.

We also note that the effect of the NNA on the doped volatile elements may contribute to solving an important missing volatile problem in geoscience, which deals with another veiled source of terrestrial volatile elements, hitherto thought to be exclusively atmosphere. The existence of volatile elements in deep Earth and the role of the deep reservoir in their cycle are still under debate. For example, a recent study discovered that a significant ratio of nitrogen comes from deep inside the Earth's crust, giving a new hint to the “nitrogen missing” problem.^[^ ^41^ ^]^ Our NNA‐driven approach implies that *hcp*‐Fe can act as a reservoir for volatile elements and contribute to their diffusion behavior so that it can be an overarching solution for the missing volatile issues and their cycle.

In this work, the entanglement of the electride and superionic state of iron‐light element alloys has been investigated. The NNAs appear at high‐pressure conditions above 100 GPa, and the H anion doped at that position becomes chemically inert, reflecting the NNA's electronically localized characteristic. This enables the non‐interacting Fe─H bond and, therefore, helps to construct the superionic environment – static Fe and freely moving H‐ions. The evolution of the superionic state was also investigated using the DFT and MD complementarily, which elucidates the overall diffusion behaviors. Finally, the effect of the secondary metal cations and the other volatile species was verified, and it was shown that the NNA‐driven phenomena depend on the major iron.

## 7 Experimental Section

All the DFT calculations were done by using the projector‐augmented wave method (PAW)^[^ ^42^, ^43^ ^]^ embedded in the Vienna ab initio simulation package (VASP).^[^ ^43^, ^44^, ^45^ ^]^ In all calculations, the generalized gradient approximation (GGA) of Perdew–Burke–Ernzerhof (PBE)^[^ ^46^ ^]^ was used for the exchange‐correlation functional with the plane‐wave cut‐off energy of 600 eV. The Monkhorts‐Pack *k*‐point mesh with 0.03∙(2π Å^−1^) and 0.02∙(2π Å^−1^) mesh resolution was used for the unit cell optimization and electronic structure calculation, respectively. At each pressure condition, the unit cell volume was optimized for both *hcp*‐Fe and *hcp*‐Fe_8_(H, C, N, or O) with the force convergence criterion of 1E‐4 eV Å^−1^. Then the electronic structure and the total energy were calculated with the self‐consistent‐field energy convergence criterion of 1E‐6 eV. The phonon band structure and the density of states were calculated by using the density functional perturbation theory (DFPT) as encoded in VASP to calculate the force constants in real space and PHONOPY.^[^ ^47^ ^]^ The Bader charge analysis is done by using the near‐grid method^[^ ^48^ ^]^ to obtain the Bader surface without lattice bias.

Ab initio molecular dynamics (AIMD) simulations were conducted to calculate the H ion diffusion behavior in the *hcp*‐Fe lattice. VASP was employed for AIMD calculations. We used the PBE exchange‐correlation functional and projector augmented wave (PAW) pseudopotentials in the calculations with an energy cutoff of 400 eV. Brillouin zone sampling was performed at the Γ point. For hydrogen bearing structure, 25 atom % hydrogen was randomly placed at the octahedral sites in the *hcp* lattice, and the supercells (4×4×2) for AIMD simulations of FeH_0.25_ contain 80 atoms. The trajectories of H anions were calculated by conducting a grid of NPT ensemble simulations at 3000 K and pressures of 15, 100, 200, and 300 GPa using a Langevin thermostat. The simulations last for 20000 steps with a step‐time of 0.5 fs.

## Conflict of Interest

The authors declare no conflict of interest.

## Author Contributions

I.P., J.H.S., and D.Y.K. conceived the research. I.P. and Y.H. carried out the main calculations. I.P., Y.H., J.H.S., and D.Y.K. analyzed the data and wrote the manuscript. All authors discussed the results and commented on the manuscript.

## Supporting information

Supporting Information

## Data Availability

The data that support the findings of this study are available in the supplementary material of this article.
