# Supplementary material for: Electride Formation of HCP‐Iron at High Pressure: Unraveling the Origin of the Superionic State of Iron‐Rich Compounds in Rocky Planets
Source: Adv Sci (Weinh). 2024 Apr 12;11(24):2308177. doi: 10.1002/advs.202308177 (PMC11200003; doi:10.1002/advs.202308177)
Supplement: Supplementary file 1 — Supporting Information [file ADVS-11-2308177-s001.pdf]

ADVANCED SCIENCE
Open Access

Supporting Information

for *Adv. Sci.*, DOI 10.1002/advs.202308177

Electride Formation of HCP-Iron at High Pressure: Unraveling the Origin of the Superionic State of Iron-Rich Compounds in Rocky Planets

Ina Park, Yu He, Ho-kwang Mao, Ji Hoon Shim and Duck Young Kim**

Supplementary Materials for

Electride Formation of HCP-Iron at High Pressure: Unraveling the Origin of the Superionic State of Iron-Rich Compounds in Rocky Planets

Ina Park *et al.*

*Corresponding author. Email: jhshim@postech.ac.kr (J.H.S.); duckyoung.kim@hpstar.ac.cn (D.Y.K.)

This file includes:

Figs. S1 to S6
Table S1, S2

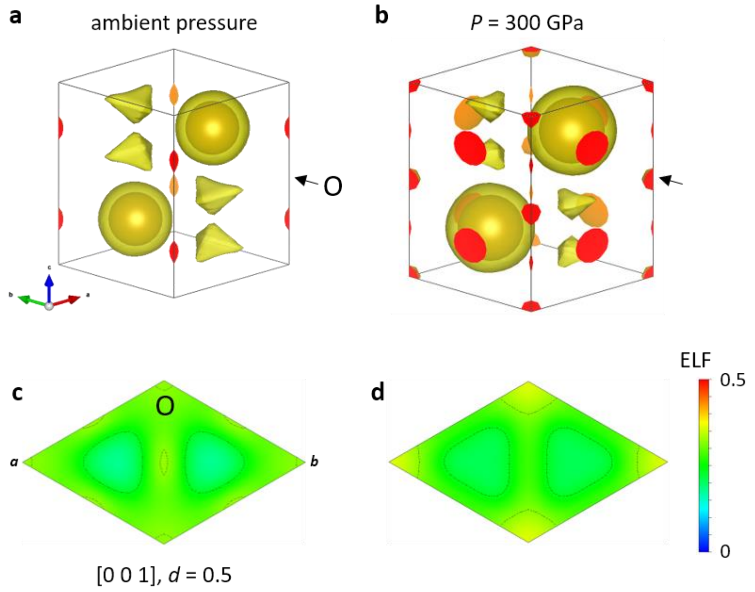

Fig. S1. Electron localization function (ELF) isosurfaces of pure hcp-Fe for **a.** ambient pressure and **b.** 300 GPa. **c,d.** ELF map and contour plot for z=0.5 plane for the same pressure conditions.

Positions	Fractional coordinates		
	x	y	z
Fe	2/3	1/3	1/4
	1/3	2/3	3/4
octahedral (O)	0	0	0
	0	0	1/2
tetrahedral (T)	2/3	1/3	1/8
	2/3	1/3	3/8
	1/3	2/3	5/8
	1/3	2/3	7/8

Table S1. **Atomic positions and interstitial positions of *hcp* iron crystal structure**

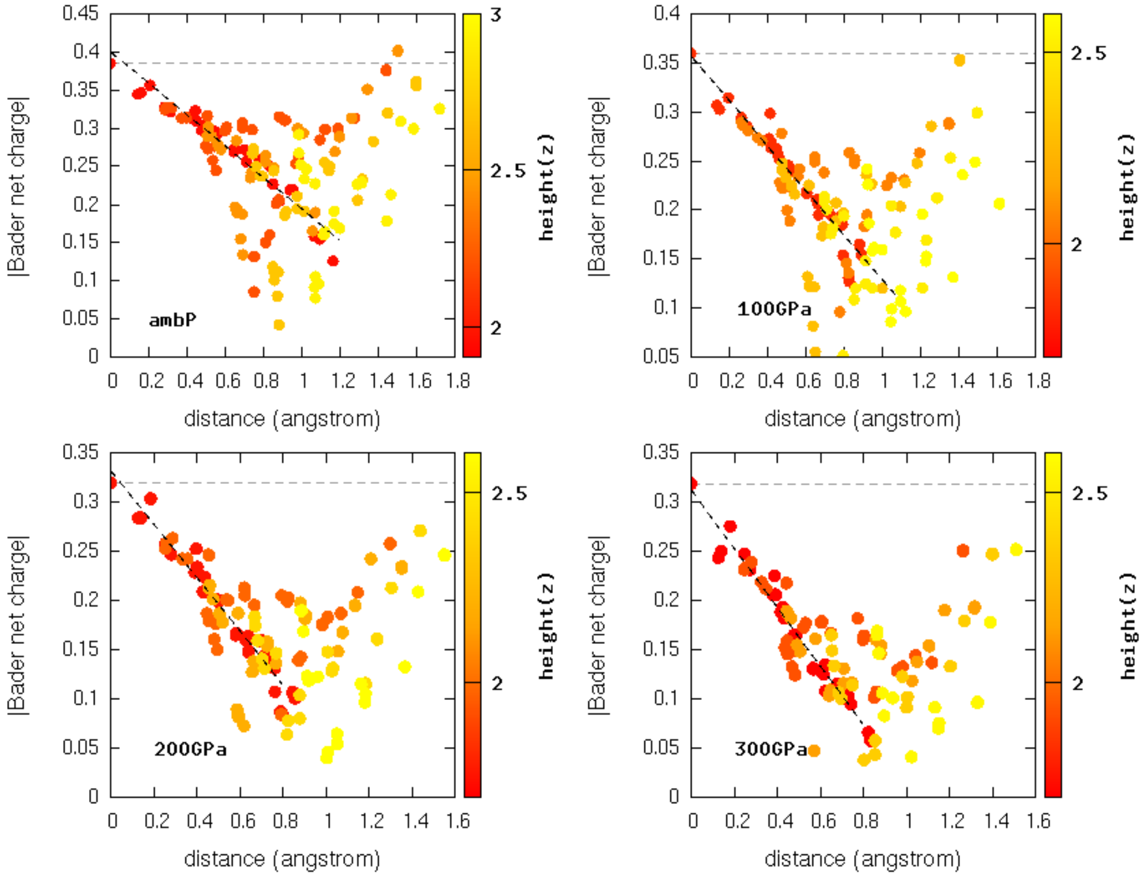

Fig. S2. Absolute Bader net charge of H anions at evenly distributed doping sites as a function of a distance from the O site at each pressure condition. The color of the dots represents the z height in Cartesian coordinates, where the red dots represent the H anions at the same height as the H^O (z_{frac} = 0.5 where the z_{frac} is the height in fractional coordinates), and the yellow dots represent the H anions at z_{frac} = 0.75. The black dashed line marks the linear fitted line for the decrease of Bader net charge of H anions at z_{frac} = 0.5 plane. The grey horizontal dashed line indicates the value at the H^O anion at each pressure condition.

To analyze the Bader net charge of H anions at every possible doping site of *hcp* iron, we generated the crystal structure of Fe_8H under the following scheme. First, we divided the range of z_{frac} from 0.5 to 0.75 in fractional coordinates into five sections. Then we created a regular mesh in the triangular space defined by the three corner positions – (0.6667, 0.8333, z_{frac}), (0.6667, 0.3333, z_{frac},), and (0.16667, 0.3333, z_{frac}) using pygalmesh python library. If the distances from Fe atoms to each mesh point exceeds 1 Å, we doped H atoms at that mesh point to create the crystal structure of Fe_8H. The calculated H positions also include one of the octahedral – (0.5, 0.5, 0.5) – and tetrahedral (0.6667, 0.3333, 0.625) – positions. All the calculated H positions are shown in Fig. S3.

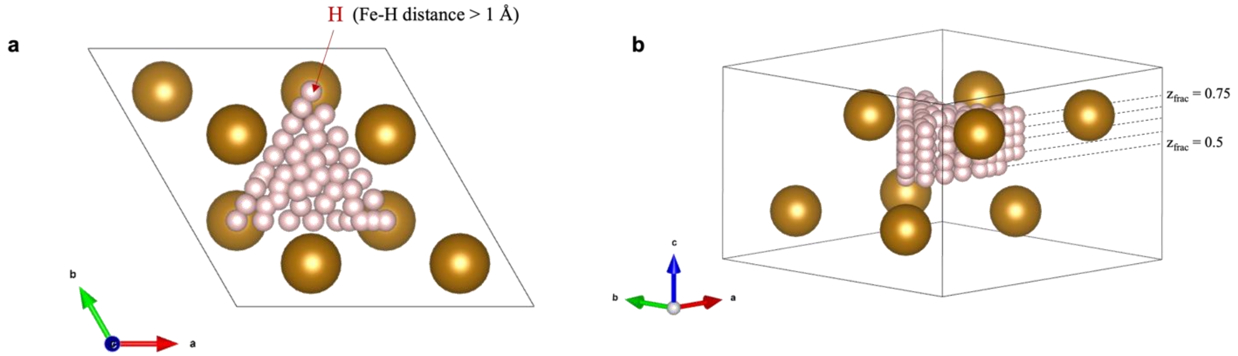

Figure S3. All calculated positions of the doped H atoms for Fe_8H. Brown is Fe atom and pink is H atom. Top view (a) and bird-eye view (b) is shown. In b, different heights (z in fractional value, z_{frac}) of H atoms are marked with dashed lines.

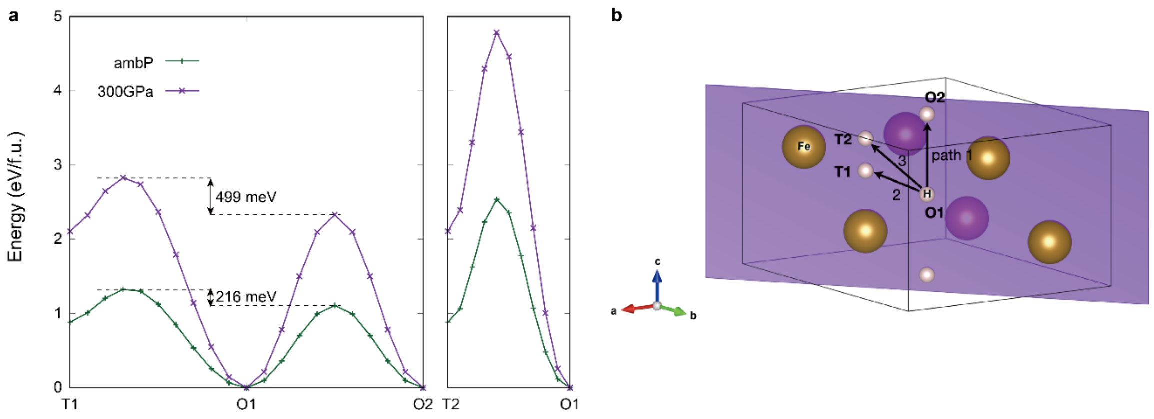

Figure S4. a. Potential energy curve for three possible diffusion paths under pressure conditions of ambient pressure and 300 GPa. The paths are depicted in **b**, with the background displaying the ELF value of (1 1 0) plane with *d* = 1.

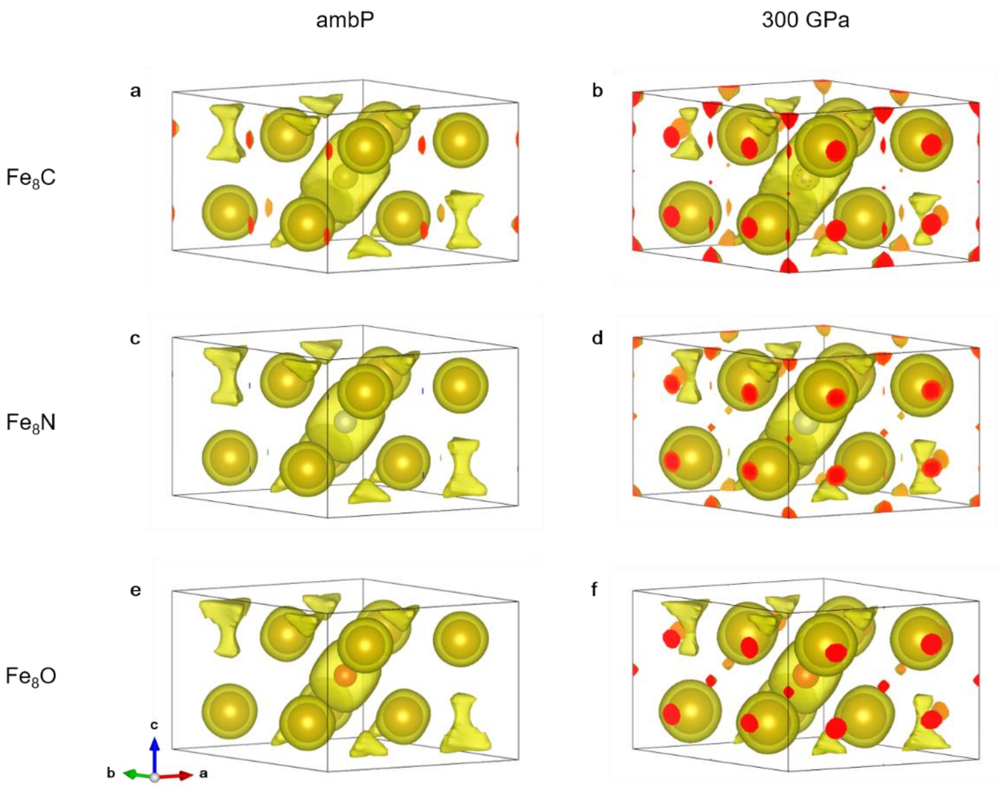

Figure S5. Electron localization function (ELF) isosurfaces with different anion element cases. **a-b.** ELF of hcp-Fe_8C at ambient pressure (ambP) (**a**) and P = 300 GPa (**b**). **c-d.** ELF of hcp-Fe_8N at ambient pressure (**c**) and P = 300 GPa (**d**). **e-f.** ELF of hcp-Fe_8O at ambient pressure (**e**) and P = 300 GPa (**f**).

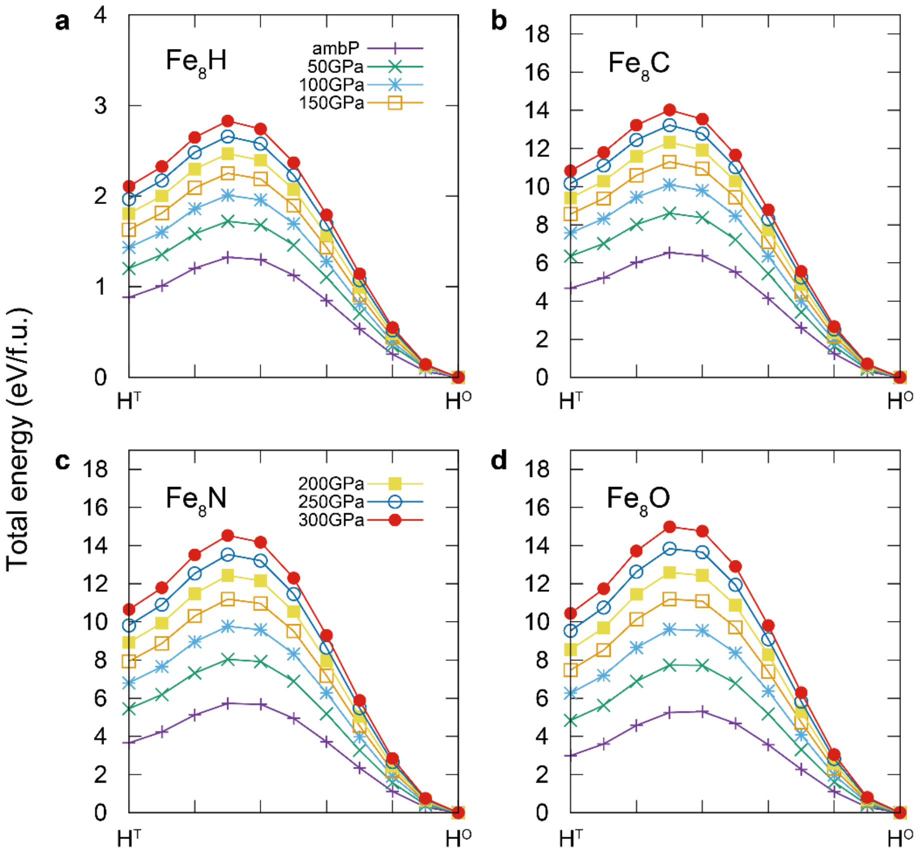

Figure S6. Potential energy curves for the linear path connecting the O and T site of *hcp*-Fe_8(H, C, N, or O) for the pressure condition from ambient pressure to P = 300 GPa.

Pressure		amb P	150 GPa	300 GPa	360 GPa
Hydrogen phase*		H_2 (*g*)	$P6_122$		
Total energy	Fe (eV/atom)	-8.278	-7.404	-6.105	-5.464
	H (eV/atom)	-3.373	-2.317	-1.748	-1.582
	Fe_8H^O (eV/f.u.)	-69.359	-61.658	-50.927	-46.141
Formation Energy (eV/f.u.)		0.235	-0.108	-0.343	-0.844

Table S2. Atomic positions and interstitial positions of *hcp* iron crystal structure

*Refer to the main text and references 35 and 36 for details.
